# Supplementary material for: Population-Level SARS-CoV-2 RT–PCR Cycle Threshold Values and Their Relationships with COVID-19 Transmission and Outcome Metrics: A Time Series Analysis Across Pandemic Years
Source: Viruses. 2025 Jan 14;17(1):103. doi: 10.3390/v17010103 (PMC11768943; doi:10.3390/v17010103)
Supplement: Supplementary file 1 [file viruses-17-00103-s001.zip › viruses-3411215-supplementary.pdf]

SUPPLEMENTARY MATERIALS

Table S1. Cross-correlations between weekly median Ct values from RT–PCR tests and epidemiological metrics related to transmission and outcomes segmented by annual time periods and predominant variant circulation.
Table S2. Lagged regression analysis of Ct values and COVID-19 transmission and outcome metrics with respect to annual time periods and predominant variant circulation.
Table S3. Summary of Granger causality and vector autoregression (VAR) analysis results by year: associ-ations between Ct values and epidemiological metrics.
Table S4. Summary of Granger causality and vector autoregression (VAR) analysis results by periods of predominant variant circulation: Associations between Ct values and epidemiological metrics.
Figure S1. Weekly SARS-CoV-2 tests reported statewide.

Table S1. Cross-correlations between weekly median Ct values from RT–PCR tests and epidemiological metrics related to transmission and outcomes segmented by annual time periods and predominant variant circulation.

Period	Metric	Lag																							
		-12	-11	-10	-9	-8	-7	-6	-5	-4	-3	-2	-1	0	1	2	3	4	5	6	7	8	9	10	11
2020-2021	SPHLJ positive SARS-CoV-2 RT-PCR tests	0.093	0.062	0.010	0.038	-0.015	-0.024	-0.019	-0.039	-0.114	-0.196	-0.282	**-0.379**	-0.236	-0.156	-0.062	-0.092	-0.167	-0.245	-0.264	-0.217	-0.098	-0.056	-0.086	-0.115
	Statewide positive SARS-CoV-2 RT-PCR tests	0.130	0.098	0.076	0.054	0.047	0.041	0.026	0.012	-0.041	-0.134	-0.238	**-0.346**	-0.296	-0.256	-0.207	-0.153	-0.157	-0.187	-0.195	-0.192	-0.179	-0.136	-0.114	-0.111
	Confirmed COVID-19 cases	0.048	0.026	-0.002	-0.018	-0.028	-0.062	-0.067	-0.082	-0.102	-0.200	-0.306	**-0.435**	-0.373	-0.275	-0.143	-0.044	-0.027	-0.089	-0.149	-0.177	-0.132	-0.047	-0.002	-0.013
	SPHLJ SARS-CoV-2 RT-PCR positivity rate	0.144	0.129	0.034	0.007	-0.058	-0.036	-0.112	-0.140	-0.194	-0.258	-0.332	**-0.481**	-0.344	-0.274	-0.118	-0.113	-0.174	-0.104	-0.176	-0.205	-0.128	-0.106	-0.017	-0.032
	Statewide SARS-CoV-2 RT-PCR positivity rate	0.097	0.079	0.082	0.042	0.044	0.080	0.073	0.058	0.039	0.004	-0.070	**-0.197**	-0.155	-0.166	-0.162	-0.131	-0.151	-0.159	-0.180	-0.154	-0.152	-0.144	-0.096	-0.079
	Statewide SARS-CoV-2 tests positivity rate*	0.182	0.171	0.167	0.130	0.137	0.164	0.167	0.162	0.148	0.115	0.043	**-0.084**	-0.049	-0.087	-0.097	-0.094	-0.114	-0.147	-0.201	-0.199	-0.214	-0.214	-0.176	-0.144
	Effective reproduction number (Rt)	0.214	0.219	0.247	0.252	0.176	0.127	0.136	0.133	0.048	-0.064	-0.146	**-0.245**	-0.165	-0.185	-0.171	-0.068	-0.079	-0.112	-0.142	-0.169	-0.105	-0.117	-0.106	-0.078
	Acute respiratory illness related hospitalizations	0.007	0.003	0.005	-0.019	-0.021	-0.028	-0.032	-0.053	-0.106	-0.192	-0.299	**-0.396**	-0.327	-0.237	-0.138	-0.044	-0.032	-0.039	-0.100	-0.104	-0.065	-0.018	-0.007	-0.031
	COVID-19 related hospitalizations	0.016	0.017	0.016	-0.008	-0.011	-0.007	-0.025	-0.053	-0.104	-0.177	-0.277	**-0.377**	-0.329	-0.245	-0.144	-0.071	-0.059	-0.058	-0.090	-0.112	-0.089	-0.041	-0.015	-0.034
	Acute respiratory illness related deaths	-0.001	-0.004	-0.026	-0.054	-0.085	-0.108	-0.147	-0.172	-0.209	-0.290	-0.325	**-0.371**	-0.216	-0.136	-0.065	-0.047	-0.076	-0.083	-0.065	-0.052	0.009	0.004	0.003	0.004
	COVID-19 related deaths	-0.002	-0.002	-0.036	-0.055	-0.091	-0.103	-0.153	-0.170	-0.224	-0.293	-0.333	**-0.366**	-0.208	-0.114	-0.051	-0.060	-0.091	-0.093	-0.063	-0.043	0.014	0.001	0.011	-0.001
2021-2022	SPHLJ positive SARS-CoV-2 RT-PCR tests	0.229	0.125	0.011	-0.069	-0.120	-0.182	-0.297	-0.339	-0.407	-0.372	-0.367	**-0.400**	-0.351	-0.282	-0.172	-0.072	0.014	0.098	0.204	0.379	0.548	0.661	0.605	0.478
	Statewide positive SARS-CoV-2 RT-PCR tests	0.273	0.229	0.138	0.026	-0.070	-0.108	-0.186	-0.267	-0.341	-0.377	**-0.383**	-0.372	-0.375	-0.360	-0.303	-0.202	-0.105	-0.016	0.062	0.148	0.298	0.467	0.601	0.597
	Confirmed COVID-19 cases	0.272	0.228	0.142	0.009	-0.091	-0.116	-0.189	-0.267	-0.332	**-0.393**	-0.385	-0.354	-0.372	-0.355	-0.312	-0.214	-0.101	0.003	0.097	0.176	0.324	0.505	0.652	0.648
	SPHLJ SARS-CoV-2 RT-PCR positivity rate	0.217	0.153	0.060	-0.063	-0.216	-0.326	-0.440	-0.480	-0.576	-0.512	**-0.591**	-0.501	-0.346	-0.279	-0.122	0.032	0.212	0.325	0.515	0.543	0.527	0.567	0.450	0.416
	Statewide SARS-CoV-2 RT-PCR positivity rate	0.335	0.308	0.231	0.115	-0.019	-0.174	-0.274	-0.384	-0.465	-0.548	**-0.578**	-0.570	-0.536	-0.458	-0.364	-0.248	-0.096	0.070	0.230	0.372	0.483	0.558	0.575	0.551
	Statewide SARS-CoV-2 tests positivity rate*	0.326	0.315	0.246	0.142	0.001	-0.183	-0.254	-0.392	-0.465	-0.564	**-0.602**	-0.573	-0.528	-0.424	-0.314	-0.182	-0.004	0.170	0.327	0.469	0.570	0.643	0.671	0.637
	Effective reproduction number (Rt)	0.079	-0.041	-0.161	-0.277	-0.357	-0.368	**-0.442**	-0.415	-0.409	-0.338	-0.250	-0.192	-0.102	0.006	0.062	0.146	0.201	0.271	0.330	0.375	0.423	0.420	0.378	0.271
	Acute respiratory illness related hospitalizations	0.218	0.143	0.053	-0.042	-0.113	-0.139	-0.236	-0.269	-0.328	**-0.333**	-0.314	-0.325	-0.302	-0.258	-0.212	-0.128	-0.071	-0.009	0.062	0.133	0.256	0.341	0.367	0.333
	COVID-19 related hospitalizations	0.223	0.151	0.060	-0.037	-0.103	-0.126	-0.223	-0.263	-0.322	-0.333	-0.317	**-0.326**	-0.314	-0.269	-0.213	-0.134	-0.063	0.007	0.078	0.173	0.305	0.402	0.445	0.395
	Acute respiratory illness related deaths	0.143	0.050	-0.019	-0.103	-0.182	-0.214	-0.315	-0.318	**-0.349**	-0.325	-0.304	-0.282	-0.211	-0.150	-0.066	-0.003	0.070	0.157	0.243	0.347	0.386	0.383	0.295	0.167
	COVID-19 related deaths	0.133	0.046	-0.025	-0.113	-0.185	-0.217	-0.317	-0.315	**-0.347**	-0.334	-0.307	-0.281	-0.207	-0.134	-0.063	0.008	0.080	0.178	0.275	0.371	0.407	0.391	0.293	0.161
2022-2023	SPHLJ positive SARS-CoV-2 RT-PCR tests	0.084	0.051	0.105	0.131	0.115	0.123	0.103	0.080	0.020	-0.075	**-0.107**	-0.087	-0.083	-0.049	-0.001	0.034	0.060	0.018	-0.040	-0.094	-0.125	-0.105	-0.067	-0.021
	Statewide positive SARS-CoV-2 RT-PCR tests	0.038	0.072	0.035	0.060	0.104	0.134	0.128	0.112	0.056	0.011	-0.049	**-0.101**	-0.090	-0.079	-0.050	0.014	0.044	0.090	0.061	-0.004	-0.069	-0.115	-0.083	-0.068
	Confirmed COVID-19 cases	0.017	0.039	0.018	0.014	0.036	0.072	0.065	0.041	-0.016	-0.049	-0.077	**-0.107**	-0.087	-0.063	-0.018	0.028	0.059	0.098	0.056	0.031	-0.010	-0.050	-0.038	-0.007
	SPHLJ SARS-CoV-2 RT-PCR positivity rate	0.176	0.101	0.162	0.168	0.137	0.112	0.093	0.053	0.007	-0.098	-0.133	-0.130	**-0.136**	-0.127	-0.122	-0.142	-0.090	-0.091	-0.137	-0.183	-0.151	-0.100	-0.057	0.005
	Statewide SARS-CoV-2 RT-PCR positivity rate	0.010	0.030	0.027	0.038	0.053	0.085	0.082	0.058	0.000	-0.058	-0.117	-0.152	**-0.159**	-0.154	-0.120	-0.107	-0.102	-0.084	-0.089	-0.097	-0.122	-0.125	-0.085	-0.062
	Statewide SARS-CoV-2 tests positivity rate*	0.044	0.052	0.039	0.042	0.047	0.073	0.067	0.030	-0.026	-0.083	-0.141	-0.183	**-0.207**	-0.222	-0.217	-0.223	-0.219	-0.194	-0.178	-0.175	-0.179	-0.156	-0.105	-0.054
	Effective reproduction number (Rt)	0.115	0.138	0.151	0.186	0.191	0.169	0.073	-0.033	-0.129	-0.174	**-0.204**	-0.190	-0.157	-0.094	-0.041	0.013	0.041	0.005	-0.048	-0.098	-0.118	-0.076	-0.026	0.097
	Acute respiratory illness related hospitalizations	0.109	0.119	0.067	0.127	0.162	0.283	0.360	0.322	0.303	0.238	0.224	**0.187**	0.185	0.190	0.090	0.112	0.129	0.044	-0.006	-0.049	-0.033	-0.042	-0.057	-0.103
	COVID-19 related hospitalizations	0.079	0.103	0.090	0.077	0.129	0.261	0.246	0.209	0.088	0.004	-0.045	**-0.093**	-0.075	-0.054	-0.063	-0.008	0.011	0.027	-0.074	-0.079	-0.124	-0.151	-0.070	-0.052
	Acute respiratory illness related deaths	0.225	0.100	0.089	0.221	0.216	0.285	0.176	-0.015	-0.035	**-0.064**	0.016	0.037	0.007	-0.090	0.055	0.080	-0.037	-0.015	-0.041	-0.174	-0.091	0.001	0.090	0.131
	COVID-19 related deaths	0.180	0.177	0.187	0.176	0.184	0.229	0.100	0.002	-0.044	**-0.133**	-0.080	-0.109	-0.082	-0.063	-0.014	0.036	-0.097	-0.112	-0.093	-0.224	-0.122	-0.065	0.080	0.172
2023-2024	SPHLJ positive SARS-CoV-2 RT-PCR tests	0.054	0.052	-0.176	-0.136	-0.206	-0.333	-0.351	-0.227	**-0.317**	-0.268	-0.274	-0.207	-0.220	-0.116	-0.196	-0.133	-0.001	-0.015	-0.077	0.040	0.064	0.069	0.269	0.125
	Statewide positive SARS-CoV-2 RT-PCR tests	0.131	0.082	0.102	0.018	-0.076	-0.156	-0.203	-0.379	-0.323	-0.391	**-0.440**	-0.338	-0.308	-0.268	-0.202	-0.152	-0.151	0.018	0.133	0.185	0.204	0.339	0.224	0.351
	Confirmed COVID-19 cases	0.138	0.093	0.060	-0.023	-0.135	-0.249	-0.315	**-0.385**	-0.331	-0.322	-0.339	-0.293	-0.271	-0.275	-0.256	-0.218	-0.185	-0.077	-0.033	-0.012	0.021	0.009	0.014	0.124

	SPHLJ SARS-CoV-2 RT-PCR positivity rate	-0.024	0.117	-0.180	-0.054	-0.051	-0.334	**-0.375**	-0.092	-0.259	-0.195	-0.163	-0.050	-0.048	0.153	-0.160	-0.037	0.106	0.160	0.102	0.208	0.121	0.028	0.224	-0.019
	Statewide SARS-CoV-2 RT-PCR positivity rate	0.284	0.269	0.195	0.181	0.086	-0.052	-0.112	-0.196	-0.332	-0.358	**-0.414**	-0.396	-0.335	-0.280	-0.210	-0.112	-0.013	0.093	0.201	0.237	0.309	0.320	0.255	0.270
	Statewide SARS-CoV-2 tests positivity rate*	0.183	0.152	0.085	0.011	-0.061	-0.140	-0.237	-0.329	-0.373	-0.405	**-0.430**	-0.406	-0.382	-0.376	-0.283	-0.263	-0.188	-0.082	-0.061	-0.053	0.053	0.066	0.089	0.100
	Effective reproduction number (Rt)	0.050	0.089	-0.063	-0.078	-0.248	**-0.284**	-0.251	-0.206	-0.228	-0.039	0.041	0.107	0.236	0.304	0.414	0.407	0.476	0.519	0.476	0.280	0.066	-0.050	-0.264	-0.226
	Acute respiratory illness related hospitalizations	0.192	0.252	0.191	0.174	0.098	**-0.127**	-0.023	0.080	-0.054	-0.017	-0.084	-0.069	-0.100	-0.006	-0.168	-0.054	0.009	0.109	0.155	0.185	0.266	0.243	0.376	0.347
	COVID-19 related hospitalizations	0.123	0.148	0.109	0.010	-0.053	-0.208	-0.314	-0.356	**-0.419**	-0.431	-0.326	-0.339	-0.308	-0.237	-0.242	-0.205	-0.211	-0.124	-0.013	0.091	0.060	0.153	0.097	0.178
	Acute respiratory illness related deaths	0.184	0.280	0.189	0.191	0.119	-0.136	0.190	-0.056	-0.115	**-0.271**	-0.097	-0.225	-0.060	-0.093	-0.193	-0.066	-0.078	0.210	0.160	0.339	0.328	0.152	0.252	0.147
	COVID-19 related deaths	0.244	0.095	0.038	0.314	-0.181	-0.231	-0.184	**-0.348**	-0.345	-0.330	-0.276	-0.170	-0.042	-0.093	0.117	0.065	0.015	0.355	0.185	0.320	0.375	0.141	0.019	0.229
Initial Lineages and Alpha and Gamma variants	SPHLJ positive SARS-CoV-2 RT-PCR tests	**-0.031**	0.081	0.122	0.156	0.166	0.161	0.154	0.134	0.130	0.123	0.126	0.136	0.155	0.043	0.010	-0.003	-0.021	-0.040	-0.047	-0.043	-0.015	-0.013	-0.022	-0.026
	Statewide positive SARS-CoV-2 RT-PCR tests	0.026	0.101	0.153	0.174	0.183	0.181	0.175	0.162	0.160	0.151	0.149	**0.147**	0.147	0.023	-0.022	-0.017	-0.019	-0.026	-0.032	-0.037	-0.032	-0.030	-0.029	-0.025
	Confirmed COVID-19 cases	**0.054**	0.113	0.155	0.171	0.180	0.175	0.174	0.166	0.153	0.144	0.131	0.122	0.129	0.013	-0.016	-0.001	0.000	-0.015	-0.032	-0.043	-0.032	-0.022	-0.017	-0.016
	SPHLJ SARS-CoV-2 RT-PCR positivity rate	**0.077**	0.179	0.249	0.256	0.287	0.301	0.276	0.275	0.241	0.253	0.117	0.170	0.283	0.074	-0.004	-0.014	-0.028	-0.026	-0.056	-0.065	-0.047	-0.063	-0.042	-0.038
	Statewide SARS-CoV-2 RT-PCR positivity rate	-0.117	-0.111	-0.090	-0.062	-0.029	-0.021	**-0.002**	0.054	0.137	0.227	0.271	0.281	0.363	0.108	0.003	0.009	0.017	0.029	0.036	0.027	0.028	0.003	-0.005	-0.008
	Statewide SARS-CoV-2 tests positivity rate*	**-0.149**	-0.142	-0.123	-0.096	-0.062	-0.055	-0.033	0.022	0.101	0.186	0.228	0.235	0.314	0.103	0.017	0.019	0.027	0.034	0.036	0.023	0.020	-0.002	-0.009	-0.010
	Effective reproduction number (Rt)	-0.080	-0.115	-0.144	-0.185	-0.258	-0.356	**-0.486**	-0.603	-0.630	-0.089	0.125	0.139	0.131	0.021	-0.24	-0.06	0.001	0.051	0.088	0.100	0.136	0.095	0.062	0.050
	Acute respiratory illness related hospitalizations	**0.019**	0.088	0.140	0.154	0.172	0.176	0.176	0.172	0.155	0.150	0.148	0.169	0.176	0.035	-0.11	0.002	0.003	0.000	-0.016	-0.023	-0.015	-0.014	-0.018	-0.023
	COVID-19 related hospitalizations	**0.028**	0.088	0.136	0.146	0.160	0.163	0.157	0.148	0.137	0.134	0.125	0.126	0.123	0.016	-0.15	-0.004	-0.003	-0.004	-0.015	-0.023	-0.018	-0.014	-0.013	-0.015
	Acute respiratory illness related deaths	**0.100**	0.150	0.147	0.146	0.144	0.132	0.123	0.106	0.118	0.109	0.127	0.138	0.151	0.035	-0.01	-0.001	-0.009	-0.013	-0.012	-0.010	0.004	-0.008	-0.012	-0.008
	COVID-19 related deaths	0.115	0.145	0.142	0.145	0.142	0.132	0.121	0.103	0.103	**0.093**	0.100	0.111	0.129	0.032	0.001	-0.004	-0.014	-0.018	-0.015	-0.010	0.004	-0.008	-0.008	-0.006
Delta variant	SPHLJ positive SARS-CoV-2 RT-PCR tests	0.081	0.090	0.076	0.010	-0.083	-0.205	-0.319	-0.353	**-0.386**	-0.361	-0.388	-0.294	-0.167	-0.115	-0.049	-0.014	0.007	0.059	0.171	0.190	0.176	0.317	0.387	0.422
	Statewide positive SARS-CoV-2 RT-PCR tests	0.094	0.093	0.078	0.034	-0.066	-0.170	-0.294	-0.343	-0.342	-0.359	**-0.366**	-0.313	-0.260	-0.202	-0.115	-0.011	-0.007	0.014	0.070	0.097	0.153	0.232	0.307	0.373
	Confirmed COVID-19 cases	0.117	0.122	0.107	0.040	-0.070	-0.165	-0.268	-0.290	-0.303	**-0.362**	-0.397	-0.326	-0.266	-0.218	-0.184	-0.149	-0.093	0.001	0.159	0.246	0.255	0.277	0.302	0.368
	SPHLJ SARS-CoV-2 RT-PCR positivity rate	0.071	0.098	0.035	-0.041	-0.210	-0.382	**-0.490**	-0.436	-0.410	-0.400	-0.383	-0.259	-0.036	-0.004	0.023	0.136	0.167	0.172	0.258	0.314	0.220	0.224	0.179	0.159
	Statewide SARS-CoV-2 RT-PCR positivity rate	0.093	0.094	0.090	0.006	-0.144	-0.273	-0.415	**-0.422**	-0.378	-0.401	-0.385	-0.296	-0.240	-0.186	-0.090	-0.008	0.103	0.204	0.265	0.328	0.329	0.315	0.293	0.243
	Statewide SARS-CoV-2 tests positivity rate*	0.112	0.112	0.104	0.021	-0.095	-0.208	-0.351	-0.365	-0.358	-0.396	**-0.398**	-0.334	-0.274	-0.228	-0.148	-0.078	0.024	0.128	0.175	0.269	0.313	0.301	0.292	0.266
	Effective reproduction number (Rt)	0.078	0.078	0.078	0.010	-0.112	-0.222	-0.368	-0.382	-0.360	**-0.393**	-0.369	-0.241	-0.153	-0.071	-0.044	-0.036	-0.005	0.049	0.147	0.178	0.189	0.272	0.372	0.443
	Acute respiratory illness related hospitalizations	0.091	0.095	0.086	0.024	-0.114	-0.215	-0.340	-0.350	-0.343	-0.378	**-0.385**	-0.277	-0.190	-0.157	-0.094	-0.050	-0.011	0.041	0.119	0.166	0.181	0.253	0.299	0.396
	COVID-19 related hospitalizations	0.100	0.106	0.095	0.029	-0.088	-0.187	-0.310	-0.333	-0.334	-0.381	**-0.397**	-0.304	-0.247	-0.195	-0.115	-0.65	-0.020	0.033	0.114	0.185	0.212	0.270	0.320	0.397
	Acute respiratory illness related deaths	0.059	0.056	0.065	-0.014	-0.146	-0.279	-0.404	**-0.420**	-0.393	-0.375	-0.351	-0.176	-0.092	-0.030	0.041	0.045	0.128	0.188	0.300	0.318	0.306	0.396	0.408	0.374
	COVID-19 related deaths	0.061	0.060	0.060	-0.020	-0.152	-0.280	-0.407	**-0.14**	-0.392	-0.397	-0.356	-0.181	-0.095	-0.002	0.044	0.057	0.138	0.205	0.320	0.339	0.326	0.387	0.402	0.349
Omicron variants	SPHLJ positive SARS-CoV-2 RT-PCR tests	0.118	0.100	0.087	0.062	0.035	-0.001	-0.038	-0.064	**-0.085**	-0.080	-0.085	-0.099	-0.084	-0.051	0.004	0.057	0.102	0.137	0.184	0.273	0.361	0.420	0.397	0.340
	Statewide positive SARS-CoV-2 RT-PCR tests	0.151	0.131	0.112	0.092	0.063	0.036	0.004	-0.033	-0.059	-0.085	**-0.093**	-0.087	-0.093	-0.093	-0.066	-0.014	0.039	0.091	0.133	0.177	0.250	0.339	0.414	0.424
	Confirmed COVID-19 cases	0.144	0.120	0.101	0.074	0.046	0.019	-0.008	-0.043	-0.067	**-0.095**	-0.090	-0.075	-0.087	-0.084	-0.059	-0.006	0.049	0.101	0.140	0.176	0.248	0.338	0.414	0.419
	SPHLJ SARS-CoV-2 RT-PCR positivity rate	0.260	0.234	0.175	0.142	0.085	-0.021	-0.087	-0.084	-0.138	-0.154	**-0.182**	-0.140	-0.099	-0.050	-0.042	0.017	0.109	0.162	0.210	0.243	0.246	0.267	0.284	0.268
	Statewide SARS-CoV-2 RT-PCR positivity rate	0.358	0.319	0.264	0.207	0.130	0.039	-0.028	-0.104	-0.79	-0.228	-0.270	**-0.280**	-0.260	-0.230	-0.184	-0.120	-0.046	0.037	0.121	0.187	0.246	0.283	0.295	0.316
	Statewide SARS-CoV-2 tests positivity rate*	0.306	0.261	0.194	0.119	0.037	-0.046	-0.132	-0.222	-0.276	-0.323	-0.356	**-0.358**	-0.344	-0.329	-0.276	-0.234	-0.164	-0.079	-0.026	0.020	0.086	0.122	0.153	0.167
	Effective reproduction number (Rt)	0.172	0.117	0.028	-0.026	-0.107	-0.163	-0.196	-0.221	**-0.238**	-0.191	-0.158	-0.116	-0.052	0.012	0.085	0.144	0.189	0.216	0.224	0.187	0.147	0.125	0.085	0.078
	Acute respiratory illness related hospitalizations	0.133	0.121	0.102	0.086	0.059	0.022	0.006	-0.018	-0.037	-0.046	-0.044	**-0.053**	-0.053	-0.014	-0.005	0.059	0.105	0.149	0.202	0.261	0.362	0.419	0.452	0.422
	COVID-19 related hospitalizations	0.094	0.073	0.065	0.041	0.024	0.007	-0.024	-0.055	-0.074	-0.085	-0.078	**-0.085**	-0.086	-0.057	-0.023	0.032	0.079	0.128	0.174	0.246	0.342	0.412	0.449	0.417
	Acute respiratory illness related deaths	0.056	0.047	0.036	0.033	0.008	-0.022	-0.038	**-0.066**	-0.057	-0.060	-0.048	-0.046	-0.001	0.036	0.095	0.149	0.192	0.270	0.345	0.438	0.473	0.475	0.419	0.339

	COVID-19 related deaths	0.035	0.026	0.018	0.011	-0.016	-0.036	-0.061	**-0.072**	-0.065	-0.068	-0.062	-0.051	-0.006	0.039	0.097	0.150	0.191	0.270	0.352	0.436	0.471	0.464	0.394	0.324

Ct: cycle threshold; SPHLJ: State Public Health Laboratory of Jalisco. Pangolin lineages corresponding to WHO variants : Initial Lineages B.1, B.1.1, B.1.1.222, B.1.1.519, Alpha vari-ant B.1.1.7, Gamma variant P.1.17, Delta variant AY.20, AY.26,and AY.100, and Omicron variant BA.1, BA.1.1, BA.2, BA.2.12.1, BA.5, JN.1, and XBB.1.5 variant.

*Includes positive RT-PCR and rapid antigen tests. Bolded values indicate the optimal lags used in the lagged regression analysis.

Table S2. Lagged regression analysis of Ct values and COVID-19 transmission and outcome metrics with respect to annual time periods and predominant variant circulation.

Metric	Period	Lag	Estimate	CI 95%lower	CI 95%upper	*p* value
SPHLJ positive SARS-CoV-2 RT-PCR tests	June 2020-May 2021	-1	-54.809	-83.516	-26.102	**<0.001**
	June 2021-May 2022	-1	-2.439	-11.059	6.180	0.572
	June 2022-May 2023	-2	-6.471	-9.934	-3.007	**<0.001**
	June 2023-May 2024	-4	-0.045	-0.419	0.329	0.809
	Initial Lineages and Alpha and Gamma variants	-12	-2.553	-26.275	21.169	0.830
	Delta variants	-4	-71.305	-141.897	-0.713	**0.048**
	Omicron variants	-4	-5.420	-16.625	5.784	0.340
Statewide positive SARS-CoV-2 RT-PCR tests	June 2020-May 2021	-1	-112.817	-213.034	-12.600	0.028
	June 2021-May 2022	-2	-15.042	-46.088	16.004	0.335
	June 2022-May 2023	-1	-8.445	-16.064	-0.826	**0.031**
	June 2023-May 2024	-2	-0.647	-1.232	-0.062	**0.031**
	Initial Lineages and Alpha and Gamma variants	-1	34.519	-21.365	90.404	0.222
	Delta variants	-2	-126.977	-265.778	11.823	0.071
	Omicron variants	-2	-8.230	-23.927	7.468	0.301
COVID-19 confirmed cases*	June 2020-May 2021	-1	-256.80	-414.51	-99.10	**0.002**
	June 2021-May 2022	-3	-150.762	-390.507	88.984	0.212
	June 2022-May 2023	-1	-65.532	-127.953	-3.111	**0.040**
	June 2023-May 2024	-5	-5.003	-10.216	0.210	0.060
	Initial Lineages and Alpha and Gamma variants	-12	17.743	-48.930	84.417	0.596
	Delta variants	-3	-694.081	-1447.609	59.447	0.069
	Omicron variants	-3	-65.986	-182.857	50.885	0.266
SPHLJ SARS-CoV-2 RT-PCR positivity rate	June 2020-May 2021	-1	-3.225	-4.718	-1.733	**<0.001**
	June 2021-May 2022	-2	-1.270	-2.075	-0.464	**0.003**
	June 2022-May 2023	0	-0.634	-1.342	0.075	0.078
	June 2023-May 2024	-6	-0.446	-0.776	-0.115	**0.009**
	Initial Lineages and Alpha and Gamma variants	-12	0.381	-0.375	1.136	0.318
	Delta variants	-6	-4.892	-8.461	-1.323	**0.010**
	Omicron variants	-2	-0.722	-1.419	-0.026	**0.042**
Statewide SARS-CoV-2 RT-PCR positivity rate	June 2020-May 2021	-1	0.286	-1.211	1.784	0.702
	June 2021-May 2022	-2	-1.281	-1.987	-0.575	**<0.001**
	June 2022-May 2023	0	-0.865	-1.567	-0.163	**0.017**

	June 2023-May 2024	-2	-0.357	-0.615	-0.099	**0.008**
	Initial Lineages and Alpha and Gamma variants	-6	-0.004	-0.725	0.717	0.991
	Delta variants	-5	-3.487	-6.693	-0.281	**0.035**
	Omicron variants	-1	-0.970	-1.561	-0.380	**0.001**
Statewide SARS-CoV-2 tests positivity rate*	June 2020-May 2021	-1	1.284	-0.680	3.247	0.195
	June 2021-May 2022	-2	-1.588	-2.340	-0.835	**<0.001**
	June 2022-May 2023	0	-1.129	-2.040	-0.219	**0.016**
	June 2023-May 2024	-2	-0.560	-1.066	-0.054	**0.031**
	Initial Lineages and Alpha and Gamma variants	-12	-0.541	-1.372	0.289	0.197
	Delta variants	-2	-2.720	-5.376	-0.065	**0.045**
	Omicron variants	-1	-1.507	-2.204	-0.810	**<0.001**
Effective reproduction number (Rt)	June 2020-May 2021	-1	-0.030	-0.104	0.044	0.419
	June 2021-May 2022	-6	-0.041	-0.070	-0.012	**0.007**
	June 2022-May 2023	-2	-0.054	-0.087	-0.021	**0.002**
	June 2023-May 2024	-7	-0.026	-0.043	-0.009	**0.004**
	Initial Lineages and Alpha and Gamma variants	-6	-0.189	-0.234	-0.144	**<0.001**
	Delta variants	-3	-0.204	-0.411	0.003	0.053
	Omicron variants	-4	-0.031	-0.053	-0.009	**0.007**
Acute respiratory illness related hospitalizations	June 2020-May 2021	-1	-82.119	-147.125	-17.114	**0.014**
	June 2021-May 2022	-3	3.807	-15.387	23.001	0.691
	June 2022-May 2023	-1	1.648	-2.140	5.436	0.386
	June 2023-May 2024	-7	-1.802	-4.062	0.457	0.115
	Initial Lineages and Alpha and Gamma variants	-12	2.888	-22.281	28.056	0.819
	Delta variants	-2	-111.915	-227.182	3.352	0.056
	Omicron variants	-1	-2.288	-9.898	5.322	0.553
COVID-19 related hospitalizations	June 2020-May 2021	-1	-62.047	-117.177	-6.916	**0.028**
	June 2021-May 2022	-1	3.350	-9.681	16.381	0.607
	June 2022-May 2023	-1	-1.725	-3.273	-0.178	**0.030**
	June 2023-May 2024	-4	-0.180	-0.500	0.139	0.260
	Initial Lineages and Alpha and Gamma variants	-12	3.079	-18.140	24.298	0.772
	Delta variants	-2	-94.620	-188.342	-0.897	**0.048**
	Omicron variants	-1	-2.801	-8.611	3.010	0.342
Acute respiratory illness related deaths	June 2020-May 2021	-1	-39.969	-72.067	-7.872	**0.016**
	June 2021-May 2022	-4	2.967	-3.882	9.816	0.387
	June 2022-May 2023	-3	-0.076	-0.784	0.633	0.830

	June 2023-May 2024	-3	-0.242	-0.596	0.111	0.173
	Initial Lineages and Alpha and Gamma variants	-12	6.095	-7.154	19.343	0.361
	Delta variants	-5	-57.255	-107.907	-6.604	0.029
	Omicron variants	-5	-1.038	-3.686	1.610	0.439
**COVID-19 related deaths**	June 2020-May 2021	-1	-36.625	-66.344	-6.906	**0.017**
	June 2021-May 2022	-4	2.366	-3.776	8.507	0.442
	June 2022-May 2023	-3	-0.186	-0.635	0.262	0.406
	June 2023-May 2024	-5	-0.145	-0.265	-0.025	**0.019**
	Initial Lineages and Alpha and Gamma variants	-3	4.579	-7.365	16.522	0.447
	Delta variants	-5	-50.452	-95.796	-5.108	**0.031**
	Omicron variants	-5	-1.000	-3.386	1.386	0.408

Pangolin lineages corresponding to WHO variants : Initial Lineages B.1, B.1.1, B.1.1.222, B.1.1.519, Alpha variant B.1.1.7, Gamma variant P.1.17, Delta variant AY.20, AY.26,and AY.100, and Omicron variant BA.1, BA.1.1, BA.2, BA.2.12.1, BA.5, JN.1, and XBB.1.5 variant.

*Includes positive RT-PCR and rapid antigen tests. Bolded values indicate the optimal lags used in the lagged regression analysis.

Table S3. Summary of Granger causality and vector autoregression (VAR) analysis results by year: associations between Ct values and epidemiological metrics.

	2020-2021					2021-2022					2022-2023					2023-2024				
	Optimal lag	Granger test *p* value	Ct lag coefficient	Ct lag *p* value	R2	Optimal lag	Granger test *p* value	Ct lag coefficient	Ct lag *p* value	R2	Optimal lag	Granger test *p* value	Ct lag coefficient	Ct lag *p* value	R2	Optimal lag	Granger test *p* value	Ct lag coefficient	Ct lag *p* value	R2
SPHLJ positive SARS-CoV-2 RT-PCR tests	1	**0.001**	-47.62	**0.001**	0.92	2	0.673	-1.92	0.451	0.98	2	**0.015**	-1.62	**0.079**	0.94	1	0.509	0.11	0.511	0.66
Statewide positive SARS-CoV-2 RT-PCR tests	3	0.375	34.15	0.264	0.95	2	0.102	-7.33	0.34	0.92	3	0.558	-0.24	0.899	0.95	3	0.118	-0.01	0.97	0.77
Confirmed COVID-19 cases	2	0.813	-8.8	0.874	0.9	2	0.143	-28.52	0.726	0.84	4	**0.036**	6.28	0.386	0.98	2	0.305	-0.59	0.599	0.91
SPHLJ SARS-CoV-2 RT-PCR positivity rate	2	**0.026**	-2.02	**0.008**	0.85	2	**0.005**	-0.71	**0.047**	0.82	1	0.489	-0.2	0.491	0.89	1	0.469	0.12	0.471	0.1
Statewide SARS-CoV-2 RT-PCR positivity rate	2	0.082	0.04	0.888	0.96	2	0.107	-0.16	0.153	0.98	3	0.087	0.07	0.338	0.99	4	0.113	-0.04	0.51	0.94
Statewide SARS-CoV-2 tests positivity rate*	2	**0.024**	-0.01	0.068	0.98	4	0.078	-0.16	0.144	0.98	2	0.58	0.02	0.761	0.99	4	0.664	-0.14	0.256	0.95
Effective reproduction number (Rt)	1	0.865	0.01	0.865	0.88	3	**0.039**	-0.01	**0.025**	0.98	2	0.441	-0.01	0.77	0.96	1	0.602	-0.01	0.603	0.63
Acute respiratory illness related hospitalizations	3	0.553	11.41	0.587	0.9	2	0.739	-2.18	0.658	0.93	1	0.708	0.5	0.709	0.85	1	0.676	0.33	0.677	0.53
COVID-19 related hospitalizations	2	0.509	17.16	0.28	0.92	2	0.544	-0.3	0.934	0.93	3	0.688	-0.57	0.339	0.93	4	0.237	-0.03	0.776	0.71
Acute respiratory illness related deaths	3	0.114	-20.15	**0.018**	0.94	2	0.203	1.21	0.418	0.96	1	0.874	0.05	0.875	0.69	1	0.381	-0.15	0.383	0.1
COVID-19 related deaths	3	0.071	-19.42	**0.011**	0.94	2	0.204	0.97	0.386	0.97	1	0.243	-0.21	0.246	0.78	2	0.342	-0.01	0.862	0.22

SPHLJ: State Public Health Laboratory of Jalisco
*Includes RT-PCR and Antigen Positive tests. Bolded values denote statistically significant results.

Table S4. Summary of Granger causality and vector autoregression (VAR) analysis results by periods of predominant variant circulation: Associations between Ct values and epidemiological metrics.

	Initial Lineages and Alpha and Gamma variants					Delta variants					Omicron variants				
	Optimal lag	Granger test *p* value	Ct lag coefficient	Ct lag *p* value	R2	Optimal lag	Granger test *p* value	Ct lag coefficient	Ct lag *p* value	R2	Optimal lag	Granger test *p* value	Ct lag coefficient	Ct lag *p* value	R2
SPHLJ positive SARS-CoV-2 RT-PCR tests	2	0.490	-4.936	0.356	0.811	1	0.155	-4.577	0.401	0.981	1	0.601	-0.950	0.418	0.960
Statewide positive SARS-CoV-2 RT-PCR tests	2	0.807	-3.886	0.802	0.721	1	0.469	-15.796	0.482	0.911	2	0.600	-2.350	0.604	0.685
Confirmed COVID-19 cases	2	0.765	-4.757	0.840	0.560	2	0.147	-80.425	0.629	0.893	2	0.511	-17.818	0.633	0.657
SPHLJ SARS-CoV-2 RT-PCR positivity rate	3	**0.006**	0.457	0.208	0.525	1	**0.009**	-0.399	0.694	0.773	2	0.264	-0.375	0.113	0.587
Statewide SARS-CoV-2 RT-PCR positivity rate	1	**0.029**	0.305	0.107	0.799	1	0.393	-0.497	0.507	0.818	1	0.289	-0.559	**0.004**	0.643
Statewide SARS-CoV-2 tests positivity rate*	1	**0.009**	0.391	0.102	0.720	1	0.210	-0.613	0.314	0.833	1	0.399	-0.970	**<0.001**	0.619
Effective reproduction number (Rt)	4	**<0.001**	-0.238	**<0.001**	0.473	3	0.181	-0.021	0.385	0.968	1	0.057	-0.008	0.443	0.223
Acute respiratory illness related hospitalizations	3	0.559	0.295	0.974	0.565	2	0.228	-2.224	0.902	0.944	3	0.442	1.753	0.077	0.934
COVID-19 related hospitalizations	3	0.706	-1.081	0.883	0.563	1	0.447	-9.254	0.542	0.913	3	0.631	-0.236	0.750	0.937
Acute respiratory illness related deaths	2	0.325	-0.782	0.863	0.572	4	0.236	-4.746	0.624	0.915	1	0.251	0.628	0.427	0.702
COVID-19 related deaths	2	0.181	-1.873	0.657	0.545	1	0.293	2.906	0.776	0.815	1	0.250	0.410	0.590	0.650

SPHLJ: State Public Health Laboratory of Jalisco

*Includes RT-PCR and Antigen Positive tests. Bolded values denote statistically significant results.

Figure S1. Weekly SARS-CoV-2 tests reported statewide.

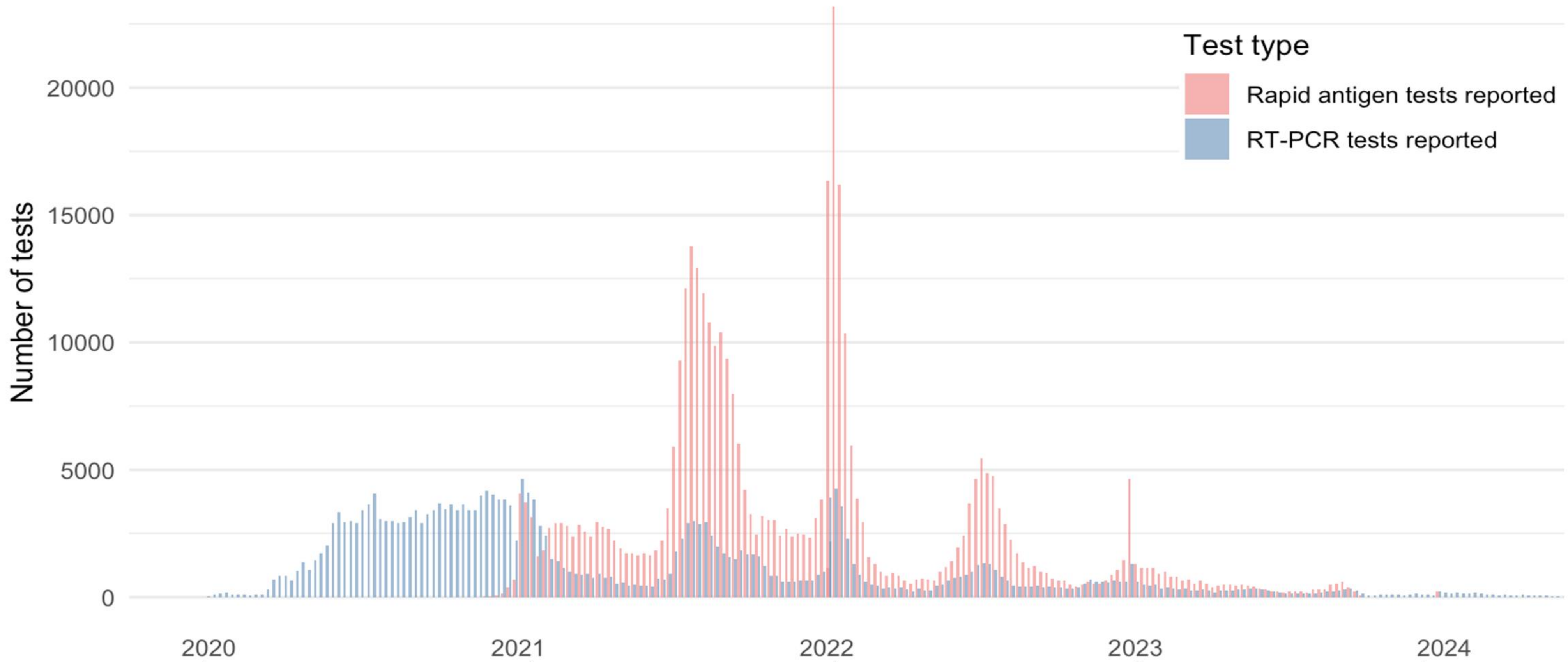

RT-PCR tests reported	120,751	77,072	42,894	12,215	2140
Rapid antigen tests reported	1298	226,665	150,387	20,586	not reported
